# Guillain-Barré syndrome with transition from hashimoto's to graves' disease: a case report

**DOI:** 10.1186/s12902-022-01067-7

**Published:** 2022-06-13

**Authors:** Mari Asano, Tsuneaki Kenzaka

**Affiliations:** 1 Department of Internal Medicine, Hyogo Prefectural Tamba Medical Center, Tamba, Japan; 2 Division of Community Medicine and Career Development, Kobe University Graduate School of Medicine, 2-1-5, Arata-cho, Hyogo-ku, Kobe, Hyogo 652-0032 Japan

**Keywords:** Hashimoto's disease, Graves' disease, Guillain-Barré syndrome, Transition, Case report

## Abstract

**Background:**

On rare occasions, there can be a transition from Hashimoto's to Graves' disease. However, there are no reported cases of transition from Hashimoto's to Graves' disease triggered by the onset of Guillain-Barré syndrome.

Case presentation.

Sixteen years prior, a 55-year-old woman was diagnosed with Hashimoto's disease and followed up without medication. One week after the appearance of signs of intestinal inflammation, weakness in the extremities was observed, and a cerebrospinal fluid test was positive for anti-GM1 IgG antibody, leading to the diagnosis of Guillain-Barré syndrome. In addition, hyperthyroidism was observed at the time of admission, and Graves' disease was diagnosed based on autoantibodies and thyroid echoes. Numbness in the extremities was relieved by high-dose intravenous gamma globulin.

**Conclusion:**

With the onset of Guillain-Barré syndrome, helper T cells became predominantly type 1, effector B cells increased in number, and thyroid-stimulating antibodies were produced, leading to the conclusion that Hashimoto's disease progressed to Graves' disease. Therefore, it is necessary to pay attention to the transition of thyroid function during Guillain-Barré syndrome.

## Background

Hashimoto's disease is a type of autoimmune disease of the thyroid gland, wherein tissue destruction accompanied by autoantibodies and lymphocyte infiltration is observed [1]. Furthermore, frequent complications of autoimmune diseases such as vitiligo and rheumatoid arthritis have been reported [1].

Graves' disease causes hyperthyroidism due to the production of autoantibodies against the thyrotropin receptor (TRAb), which increases thyroid hormone synthesis and secretion [2]. It causes various symptoms such as weight loss, tiredness, palpitations, dyspnea, and tremors [2].

Hashimoto's disease rarely shifts to Graves' disease. When it happens, it is due to helper T cells becoming mostly type 1 (Th1), increasing effector B cell numbers, and production of thyroid-stimulating antibodies (TSAb) [3].

Here, we report a case of transition from Hashimoto's to Graves' disease triggered by Guillain-Barré syndrome.

### Case presentation

Sixteen years prior, a 55-year-old woman was diagnosed with Hashimoto's disease and followed up without treatment. At that time, the patient’s diagnosis was based on anti-thyroglobulin antibody (Ab) + , anti-thyroid peroxidase (TPO)Ab + , TSH receptor Ab − , TSAb − , and thyroid echo.

She had fever, vomiting, and diarrhea 17 days before her visit and was treated for acute enteritis by her primary care physician. The symptoms disappeared for approximately 1 week. No culture tests were performed. Ten days before the visit, weakness of the extremities and sensory impairment were observed. Simultaneously, she was referred to our hospital because of sweating and finger tremor. Her medical history included hypertension, but no history of drinking or smoking, and treatment with valsartan 40 mg/day and amlodipine besilate (5 mg/day) for 8 years.

During the initial physical examination on admission, the following vital signs were noted: consciousness, clear; temperature, 36.9 ºC; blood pressure, 150/75 mmHg; pulse rate, 112 beats/min; respiration rate, 12 breaths/min; and peripheral capillary oxygen saturation, 98% in room air.

Her height was 161 cm, weight was 48.6 kg, and body mass index 18.7. Eye movement was normal, there was no exophthalmos, and although the thyroid gland was swollen, it was soft, and there was no tenderness. Neurological findings revealed no abnormalities in the cranial nerve system. In the manual muscle test (MMT), the biceps brachii/triceps and iliopsoas muscles and the quadriceps femoris/flexor muscle group scores decreased to 3. Regarding sensory impairment, glove-sock-type hand and foot sensory impairments were observed in the sense of touch, pain, and temperature, but no weakening of the tendon reflex was observed.

**Fig. 1:**
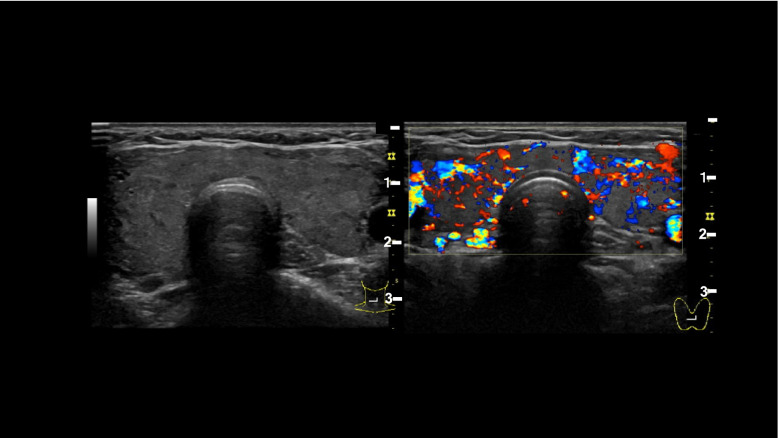
Thyroid ultrasonography. Thyroid ultrasonography revealed diffuse swelling of the thyroid gland and abundant blood flow

Her initial blood workup results were as follows: level of TSH < 0.003 μIU/mL (reference value 0.50–5.00 μIU/mL), FT4 6.07 ng/dL (reference value 0.90–1.70 ng/dL), and FT3 17.57 pg/mL (reference value 2.30–4.00 pg/mL). Blood gamma globulin levels were normal, as were ammonia and vitamin B12 levels. Mycoplasma Ab, CMV-IgG, and IgM all tested negative (Table 1). Urinalysis and cerebrospinal fluid (CSF) test results were normal, and stool culture showed only normal bacterial flora. Nerve conduction studies revealed no abnormal findings. In additional blood tests, level of anti-thyroglobulin Ab was 260.0 IU/mL (standard value 0.0–27.9 IU/mL), anti-TPO antibody was ≥ 600 IU/mL (standard value 0.0–15.9 IU/mL), TSH receptor Ab was 9.0 IU/L (standard values were 0.0–1.9 IU/L), and TSAb was 1719% (standard values 0–120%), all elevated. Thyroid ultrasonography revealed diffuse swelling of the thyroid gland and abundant blood flow (Fig. 1). CSF examination revealed no protein cell dissociation at a cell count of 3/μL and protein level of 31 mg/dL. Blood and CSF culture test results were negative. Head MRI revealed no abnormal signals in the brain.

**Table 1:** Laboratory data upon admission

Parameter	Recorded value	Standard value
White blood cell count	3590/µL	4500–7500/µL
Neutrophils	45.1%	42–74%
Lymphocytes	44.3%	18–50%
Hemoglobin	12.8 g/dL	11.3–15.2 g/dL
Platelet count	39 × 10^4^/µL	13–35 × 10^3^/µL
Thyroid stimulating hormone	< 0.003 μIU/mL	0.5–5.0 μIU/mL
Free thyroxine	6.07 ng/dL	2.30–4.00 ng/dL
Free triiodothyronine	17.57 pg/mL	0.90–1.70 pg/mL
C-reactive protein	0.02 mg/L	≤ 0.60 mg/dL
Total protein	6.7 g/dL	6.9–8.4 g/dL
Albumin	3.6 g/dL	3.9–5.1 g/dL
Total bilirubin	0.7 mg/dL	0.2–1.2 mg/dL
Aspartate aminotransferase	51 U/L	11–30 U/L
Alanine aminotransferase	66 U/L	4–30 U/L
Lactase dehydrogenase	192 U/L	109–216 U/L
Creatine kinase	95 U/L	40–150 U/L
Blood urea nitrogen	12.5 mg/dL	8–20 mg/dL
Creatinine	0.45 mg/dL	0.63–1.03 mg/dL
Sodium	140 mEq/L	136–148 mEq/L
Potassium	3.8 mEq/L	3.6–5.0 mEq/L
Chloride	107 mEq/L	98–108 mEq/L
Corrected calcium	10 mg/dL	8.5–10.2 mg/dL
Phosphoric acid	3.2 mg/dL	2.5–4.5 mg/dL
Magnesium	1.89 mg/dL	1.8–2.6 mg/dL
Glucose	116 mg/dL	70–109 mg/dL
Hemoglobin A1c	5.7%	5.6–5.9%

Weakness and sensory deficits in the extremities after acute enteritis were thought to be symptoms of Guillain-Barré syndrome. In addition, periodic paralysis associated with hyperthyroidism was considered a differential diagnosis.

With respect to treatment, from the first day of hospitalization, gamma globulin was administered at 400 mg/kg/day for 5 days. On the 3rd day, the MMT score had improved to 4, and on the 7th day, it had improved to 5. Hyperthyroidism was treated with mercazole 30 mg/day. FT4 levels decreased to 2.41 ng/dL on the 8th day. Although the MMT score improved, hyperthyroidism persisted, and we believe that the limb weakness and sensory impairment were not due to periodic paralysis. An anti-glycolipid antibody test of the CSF was positive for anti-GM1 IgGAb, and limb muscle weakness and sensory impairment were diagnosed as symptoms of Guillain-Barré syndrome. Both the weakness and sensory impairment were completely cured thereafter, and she was discharged on the 12th day of hospitalization.

On the 18th day after onset, the FT4 level decreased to 1.52 ng/dL. Since then (currently 2.5 years), the patient has reported no muscle weakness or sensory impairment. Mercazole was gradually reduced and discontinued after 2 years. Thyroid function is currently normal. An outpatient follow-up period of 2.5 years has passed since then. In the last follow-up visit, level of anti-thyroglobulin Ab was 54.2 IU/mL, anti-TPO Ab 207.0 IU/mL, TSH receptor Ab < 0.8 IU/L, TSAb 143%, TSH 0.3809 μIU/mL, FT4 0.97 ng/dL, and FT3 2.77 pg/mL, all of which are consistent with the course of Graves' disease.

## Discussion and conclusion

We present a case of Hashimoto's disease evolving to Graves' disease in the wake of Guillain-Barré syndrome; in rare cases, Hashimoto's and Graves' disease are associated with Guillain-Barré syndrome. To the best of our knowledge, this is the third report of Guillain-Barré syndrome in Graves' disease worldwide.

Furthermore, only six cases of Guillain-Barré syndrome or its subtype, Miller Fisher syndrome, with thyroid disease complications have been reported (Table 2) [4–8]. The median age of affected patients was 53.5 years (7–70 years), and all six patients were women. There was one case of Graves' disease and one of hyperthyroidism (no description of Graves' disease) as thyroid disease. With respect to geographical location, two patients were Japanese, three Italian, and one Swiss. In treating Guillain-Barré/Miller Fisher syndrome, gamma globulin was administered in four cases and steroid treatment in two cases. Very rarely, autoantibody-related thyroid disease can be associated with Guillain-Barré syndrome, which also involves autoantibodies.

**Table 2:** Reports of thyroid disease complications and Guillain-Barré syndrome

Case	References	Age Sex	Race		Thyroid disease complications	Treatment
1	4, Polizzi A, et al	7/F	Italian	GBS	Hashimoto's disease	Gamma globulin administration
2	4, Polizzi A, et al	42/F	Italian	MFS	Hyperthyroidism	Steroid administration
3	5, Kagiyama T, et al	53/F	Japanese	GBS	Hashimoto's disease, Hashimoto's encephalopathy	Gamma globulin administration
4	6, Shakado S, et al	54/F	Japanese	GBS	Graves' disease	Immune adsorption therapy, Gamma globulin administration
5	7, Pandolfi C, et al	56/F	Italian	GBS	Hypothyroidism	Steroid administration, Plasmapheresis
6	8, Vetsch G, et al	70/F	Swiss	MFS	Hashimoto's disease	Gamma globulin administration
Present case		55/F	Japanese		Hashimoto's ⇒ Graves' disease	Gamma globulin administration

*F* Female, *M* Male, *GBS* Guillain-Barré syndrome *MFS* Miller Fisher syndrome

The transition mechanism from Hashimoto's to Graves' disease is through the dominance of Th1 cells, increased effector B cells, and TSAb production [2, 3]. In addition, helper T cells are predominantly Th17, and this increase correlates with goiter enlargement and TSAb [2, 3]. On the other hand, Th1 and Th17 become dominant during the onset of Guillain-Barré syndrome [9]. In this case, the onset of Guillain-Barré syndrome increased Th1 and Th17, possibly leading to Hashimoto's disease transitioning into Graves' disease due to the production of TSAb. However, the issue of whether Graves’ disease is biased toward the Th1 or Th2 functional subset remains controversial [2].

As an alternative hypothesis, Graves' disease can be triggered by viral infection [10]. It is possible that an unidentified pathogen could have caused both Guillain-Barré syndrome and Graves' disease simultaneously. However, it is highly unlikely that a viral infection triggered both Guillain-Barré syndrome and Graves' disease concurrently.

Patients with autoimmune thyroid disorders (i.e., Hashimoto's disease and Graves' disease) often have multiple autoimmune diseases [11]. This results from a complex interaction between genetic predisposition and environmental/endogenous factors [11]. In particular, it has been reported that multiple autoimmune diseases are more predominant in adult patients than in pediatric/adolescent patients [12]. In the current case, it is assumed that the patient had an original predisposition to Hashimoto's disease and had endogenous factors predisposing her to Guillain-Barré syndrome, a type of autoimmune disease.

In conclusion, this report presents a case wherein Hashimoto's disease changed into Graves' disease as a result of Guillain-Barré syndrome. Although rare, Guillain-Barré syndrome can develop in patients with thyroid disease. If a patient with Hashimoto's disease develops Guillain-Barré syndrome, it may progress to Graves' disease; thus, it is necessary to pay attention to the transition of thyroid function during the course of Guillain-Barré syndrome.

## Data Availability

All data generated or analyzed during this study are included in this published article.

## Abbreviations

Ab: Antibody
TRAb: Autoantibodies to the thyrotropin receptor
TSAb: Thyroid stimulating antibody
MMT: Manual muscle test
CSF: Cerebrospinal fluid

## References

[1] Fallahi P, Ferrari SM, Ruffilli I, Elia G, Biricotti M, Vita R (2016). The association of other autoimmune diseases in patients with autoimmune thyroiditis: Review of the literature and report of a large series of patients. Autoimmun Rev.

[2] Smith TJ, Hegedüs L (2016). Graves' disease. N Engl J Med.

[3] Aniszewski JP, Valyasevi RW, Bahn RS (2000). Relationship between disease duration and predominant orbital T cell subset in Graves' ophthalmopathy. J Clin Endocrinol Metab.

[4] Polizzi A, Ruggieri M, Vecchio I, Genovese S, Rampello L, Raffaele R (2001). Autoimmune thyroiditis and acquired demyelinating polyradiculoneuropathy. Clin Neurol Neurosurg.

[5] Kagiyama T, Yokoyama Y, Watanabe Y, Yokoyama N, Sayake T, Ibayashi S (2015). A case of Hashimoto's encephalopathy after Guillain-Barre syndrome (GBS). Jpn J Rehabil Med.

[6] Shakado S, Fujino T, Tsugawa J, Eto S, Sugimoto M, Kadomitsu S (2005). A case of exacerbation of Graves' disease and Guillain-Barre syndrome after Campylobacter enteritis. Rinsho Kenkyu.

[7] Pandolfi C, Filippi C (1989). Guillain-Barré syndrome associated with hypothyroidism. Report of a case Minerva Med.

[8] Vetsch G, Pato U, Fischli S, Lepper S, Lönnfors-Weitzel T, Offinger A (2008). A combined presentation of Graves' disease and Miller-Fisher syndrome. Lancet.

[9] Zhang HL, Zheng XY, Zhu J (2013). Th1/Th2/Th17/Treg cytokines in Guillain-Barré syndrome and experimental autoimmune neuritis. Cytokine Growth Factor Rev.

[10] Larouche V, Tamilia M (2017). Cytomegalovirus-mononucleosis-induced thyroiditis in an immunocompetent patient. Endocrinol Diabetes Metab Case Rep.

[11] Ruggeri RM, Giuffrida G, Campennì A (2018). Autoimmune endocrine diseases. Minerva Endocrinol.

[12] Ruggeri RM, Trimarchi F, Giuffrida G, Certo R, Cama E, Campennì A (2017). Autoimmune comorbidities in Hashimoto's thyroiditis: Different patterns of association in adulthood and childhood/adolescence. Eur J Endocrinol.

